# Development and Validation of the 34-Item Disability Screening Questionnaire (DSQ-34) for Use in Low and Middle Income Countries Epidemiological and Development Surveys

**DOI:** 10.1371/journal.pone.0143610

**Published:** 2015-12-02

**Authors:** Jean-François Trani, Ganesh Muneshwar Babulal, Parul Bakhshi

**Affiliations:** 1 Brown School and Institute of Public Health, Washington University in St Louis, St. Louis, Missouri, United States of America; 2 Department of Neurology, Washington University School of Medicine in St Louis, St. Louis, Missouri, United States of America; 3 Program in Occupational Therapy, Washington University School of Medicine in St Louis, St. Louis, Missouri, United States of America; University of Otago, NEW ZEALAND

## Abstract

**Background:**

Although 80% of persons with disabilities live in low and middle-income countries, there is still a lack of comprehensive, cross-culturally validated tools to identify persons facing activity limitations and functioning difficulties in these settings. In absence of such a tool, disability estimates vary considerably according to the methodology used, and policies are based on unreliable estimates.

**Methods and Findings:**

The Disability Screening Questionnaire composed of 27 items (DSQ-27) was initially designed by a group of international experts in survey development and disability in Afghanistan for a national survey. Items were selected based on major domains of activity limitations and functioning difficulties linked to an impairment as defined by the International Classification of Functioning, Disability and Health. Face, content and construct validity, as well as sensitivity and specificity were examined. Based on the results obtained, the tool was subsequently refined and expanded to 34 items, tested and validated in Darfur, Sudan. Internal consistency for the total DSQ-34 using a raw and standardized Cronbach’s Alpha and within each domain using a standardized Cronbach’s Alpha was examined in the Asian context (India and Nepal). Exploratory factor analysis (EFA) using principal axis factoring (PAF) evaluated the lowest number of factors to account for the common variance among the questions in the screen. Test-retest reliability was determined by calculating intraclass correlation (ICC) and inter-rater reliability by calculating the kappa statistic; results were checked using Bland-Altman plots. The DSQ-34 was further tested for standard error of measurement (SEM) and for the minimum detectable change (MDC). Good internal consistency was indicated by Cronbach’s Alpha of 0.83/0.82 for India and 0.76/0.78 for Nepal. We confirmed our assumption for EFA using the Kaiser-Meyer-Olkin measure of sampling well above the accepted cutoff of 0.40 for India (0.82) and Nepal (0.82). The criteria for Bartlett’s test of sphericity were also met for both India (< .001) and Nepal (< .001). Estimates of reliability from the two countries reached acceptable levels of ICC of 0.75 (p<0.001) for India of 0.77 for Nepal (p<0.001) and good strength of agreement for weighted kappa (respectively 0.77 and 0.79). The SEM/MDC was 0.80/2.22 for India and 0.96/2.66 for Nepal indicating a smaller amount of measurement error in the screen.

**Conclusions:**

In Nepal and India, the DSQ-34 shows strong psychometric properties that indicate that it effectively discriminates between persons with and without disabilities. This instrument can be used in association with other instruments for the purpose of comparing health outcomes of persons with and without disabilities in LMICs.

## Introduction

Despite recent attempts to measure disability in low and middle-income countries (LMICs), there is a lack of comprehensive, accessible and cross-culturally validated tools to identify persons facing activity limitations, functioning difficulties and participation restrictions as defined in the International Classification of Functioning, Disability and Health (ICF) [1–4]. Different approaches to measuring disability have resulted in a wide variety of prevalence estimates. For instance, a recent study exploring adult disability in 49 countries -33 LMICs and 16 upper middle and high income countries—using the health state description module of the World Health Survey (WHS)—found estimates varying from 3.3% and 4.6% respectively in Malaysia and Vietnam to 28.1% in the Russian Federation [5]. Another study using four questions from the WHS found severe disability prevalence estimates in 54 countries varying from 0.3% and 0.4% in Luxembourg and Uruguay to 15.2% in South Africa [6]. The wide gap observed across countries can be explained by several factors: cultural differences in perception of disability, variability in study design, modalities of implementation of survey (including training of the data collection team) and a clear purpose of the disability measure (e.g. informing policy makers or providing medical rehabilitation) [7]. Nevertheless, some of the variation has also been attributed to the tool’s capacity to identify persons with disability across different cultural contexts [8, 9].

The 2013 United Nations General Assembly on Disability reiterated the goal to improve disability data collection as stipulated by article 31 of the United Nations Convention on the Rights of Persons with Disabilities (UNCRPD). Disability is a complex concept that associates individual impairment, limitations in activities and functioning difficulties but also restriction in the individual’s participation in society [10]. Measuring disability requires designing complex questionnaires that pursue various objectives. The 34-item disability-screening questionnaire (DSQ-34, S1 File) is complementary to initiatives like the WHODAS-2 36-item scale, the WG short set of and extended questions for core functional domains, the Rapid Assessment of Disability (RAD) and the World Health organization Model Disability Survey (MDS). Our approach differs from existing instrument-development methodologies. First, the rationale was to identify activity limitations and functioning difficulties in LMICs where the health system is not equipped to establish in-depth assessments. We examined if persons identified with activity limitations and functioning difficulties could benefit from access to services that meet their needs. We further investigated level of poverty and barriers linked to stigma and discrimination, and defined strategies to foster social inclusion. Achieving these analyses requires the use of complementary instruments adapted for complex investigations, tailored to the political, social and cultural context, focusing on the aim of the study, identifying needs and opportunities as well as barriers individuals face to fully participate in society [11–13]. Unlike the MDS, the DSQ-34 alone does not capture aspects of participation in a given environment except when used alongside instruments that identify issues that create a disabling situation [14]. In fact, the DSQ-34 was used with a questionnaire built on the capability approach defined by Amartya Sen with contextual adaptation depending on the scope of the study: measuring overall wellbeing, social inclusion, poverty, needs, use and barriers to use of services [12].The DSQ-34 is designed to work in association with specific instruments that investigate disability defined following Trani and Bakhshi (2011) as “the condition that results from the interaction between an individual impairment in functioning and the community and social resources, beliefs and practices that enable or prevent a person from participating in all spheres of social life and taking decisions that are relevant to his/her own future” [12] p. 49. It can also be used in LMIC contexts with other instruments to explore access to services, multidimensional poverty, or social participation based on the defined aims of a given study. The WHODAS-2 36-item scale and the WG evaluate participation and barriers to participation, negative attitudes and wellbeing, but with a limited set of questions. Similar measures that assess community access and disability impact (RAD) [4] and environmental factors that facilitate or hinder participation (MDS) [15] use the same items whatever the context and cannot adapt to specific, contextually defined, policy objectives.

The DSQ-34’s purpose was to identify types and levels of activity limitations and functioning difficulties. We argue that only the MDS provide sufficient details about various aspects of activity limitations and functioning difficulties experienced by individuals with the most stigmatized forms of impairments such as mental disorders.

Initial development of the screening tool began in Afghanistan. A second phase was conducted to improve sensitivity to further detect activity limitations and functioning difficulties. A third and final stage sought to establish reliability and validity. The purpose of this paper is to present the stages of development of the tool in Afghanistan, the refinement process of the tool in Darfur and analyze the psychometric properties obtained from surveys in India and Nepal.

## Methods

Information relating to content, scoring and development of the DSQ in Afghanistan are available in S2 File.

### Data collection in India and Nepal

The New Delhi, India survey was conducted between November 2011 and June 2012. Patients (N = 649) diagnosed with either schizophrenia or severe affective disorders were informed about the survey by one of 10 treating psychiatrists of the Department of Psychiatry of the Dr Ram Manohar Lohia Hospital (RML). The inclusion criteria were a diagnosis of severe mental illness made by a psychiatrist based on the ICD 10 criteria and ability to provide informed consent. A non-psychiatrically ill comparison group (N = 647) was randomly selected and matched to patients on gender, age and neighborhood of residence. A total of 5289 (2551 cases; 2738 controls) household members aged 15 and above were screened for disability. Test-retest was carried out with a gap of 10 to 15 days by the same data collectors. The inter-rater reliability checks were carried out on the same day by two different data collectors. Two experienced supervisors and 10 Masters-level students were trained over a period of two weeks. Data collectors were trained on survey concepts and goals (1 day), mental illness issues and awareness (1 day), interview techniques (8 days including item by item explanation of instruments) followed by review, test, and debriefing. Role-play and field practice interviews were organized. Four investigators observed 200 (15.4%) interviews.

The Nepal study was undertaken in Makwanpur district, in central Nepal between October 2010 and April 2011. Women with disabilities were identified using the DSQ-34 within a cohort of 13,637 married women (aged between 17 and 65) from 30 population clusters enrolled in a randomized controlled assessment of participatory women’s groups on birth outcomes. Women were screened for the same six types of disabilities: motor/physical, sensory, learning/developmental, behavioral, mood/affect, and neurological. A sample of 53 women was interviewed twice in the same day by different data collectors for inter-rater reliability, and again after an interval of 10–14 days for test-retest reliability. Local investigators (14) received a two-day training on the instrument and the methodology and completed 100 interviews to validate the questionnaire. Local investigators also trained and supervised 49 local data collectors and observed 740 (5.3%) interviews.

### Statistic analysis

Data distributions were visually checked using frequencies, scatterplots and histograms. Surveys with missing responses were excluded from all analyses (0.3% and 0.1% in India and Nepal). Socio-demographic variables were calculated using descriptive statistics. We assessed sensitivity and specificity of the cutoff for moderate, severe and very severe disability for the initial 27-item tool in Afghanistan comparing it to a 46-item tool. Sensitivity was calculated as the proportion of respondents screened as disabled by the 27 items—and also identified by the 46-item screening tool as disabled. Specificity was calculated as the proportion of people identified as non-disabled by the DSQ-27 and also identified as non-disabled by the second tool. We used established cutoffs based on Terwee et al. (2007: 36) where threshold of 75% was consider as being a “positive rating”.

Internal consistency for the total scale was calculated using raw and standardized Cronbach’s Alpha. Internal consistency within each domain was tested using a standardized Cronbach’s Alpha. Exploratory factor analysis (EFA) using principal axis factoring (PAF) evaluated the lowest number of factors to account for the common variance among the questions in the screen. Unlike the more commonly used principal component analysis, PAF is able to model error variance. Test-retest reliability was determined by calculating intra-class correlation (ICC) to assess the correlation between the total score between the two time points. Inter-rater reliability was determined by the kappa statistic, which assessed the level of agreement between the two raters, followed up using Bland-Altman plots to corroborate the results. Reliability, specifically dispersion of measurement errors was calculated using the standard error of measurement formula and the smallest change considered to be significant was calculated with the minimal detectable change. Finally, the ability of the DSQ-34 to detect disability was evaluated by a t-test with cases and controls in India. Chi-square analyses assessed differences in gender, education (below high school vs. high school or beyond) and marital status (married vs. unmarried) while t-tests assessed group differences in age and monthly income. All analyses were completed on a Macintosh computer using SPSS version 21 (Chicago, Illinois, USA) and Stata 13 (College Station, Texas, USA) to ensure accuracy across both statistical packages.

### Ethics

Consent was obtained for adults and assent was obtained from the guardian of children below 18 years old enrolled in the studies. Illiterate respondents provided verbal consent in the presence of a third party. Initial development of the disability screening questionnaire and the consent procedure received ethical approval from the Committee on Human Research of the Johns Hopkins Bloomberg School of Public Health and from the Ministry of Public Health of Afghanistan. All later stages of development and the consent procedure received approval from the University College London Research Ethics Committee and the Dr Ram Manohar Lohia Hospital Institutional Ethics Committee in India and the Nepal Health Research Council.

## Results

### Participant demographics

In Afghanistan, we interviewed a sample of 2696 respondents, 1011 (37.5%) with moderate, severe, or very severe disability and 1685 (62.5%) without disability. Males were slightly over represented (1517; 56.3%), as were adults over 15 (1860; 69.0%). Among adults, 77.3% of non-disabled men (7.9% of women) were working compared to 47.5% of disabled men (2.7% of women). Education was extremely low, particularly for girls and women with disabilities (10.2% went to primary school).

In India, the mean age of respondents was 36.3 years with close to an even distribution between genders (47.4% female). Approximately 80% of the sample had below high school education while nearly half of the sample was married. The mean household monthly income (INR) was 19436 ($298.1); approximately 66% of the household heads were employed in some form of occupation that provided income (see Table 1).

**Table 1:** Respondents’ characteristics in India and Nepal.

Demographics	India	Nepal
Sample, (n)	5289	13637
Age, (mean (SD), range)	36.3 (15.9) 15–96	31.97 (7.1) 17–65
Sex (female) (%)	47.4%	100.0%
Disability Severity (%)	Non disabled– 84,4%	Non disabled—68.2%
	Mild disabled– 6.2%	Mild disabled– 14.4%
	Moderate disabled– 5.5%	Moderate disabled– 10.8%
	Severe disabled– 3.10%	Severe disabled 5.1%
	Very severe disabled– 0.78%	Very severe disabled 1.4%
Education (%)	Not literate– 10.7%	School grade attained
	Literate without formal– 3.5%	None– 66.4%
	Literature, below primary– 2.5%	Grade 1 to 6–22.0%
	Primary– 6.9%	Grade 7 to 8–5.6%
	Middle school– 36.5%	Grade 9 to 12–6.0%
	Secondary– 19.6%	Literacy
	Diploma– 0.8%	Unable to read– 56.3%
	Graduate– 15.5%	Read with difficulty– 16.4%
	Post Graduate– 3.8%	Read with ease– 27.3%
Marital Status (%)	Unmarried– 33.0%	Married– 100.0%
	Married– 59.9%	
	Separated– 0.7%	
	Divorced– 0.9%	
	Widowed– 5.5%	
Household income (currency)	Monthly income (Indian Rupees-INR) 19,436 (26,614.0) 0–480,000	Livestock (Nepalese Rupees-NR) 520,573 (13,957,829.2) 0–1.0E+8
Main occupation of the Household Head (%)	Work on own farm—0.1%	Agriculture– 90.3%
	Self employed (home-based work)– 3.4%	Waged labor– 5.0%
	Self employed (workplace outside home)– 8.5%	Salaried or government– 2.3%
	Works as regular wage/salaried– 23.9%	Small business– 2.2%
	Casual Agricultural laborer– 0.2%	Other– 0.2%
	Casual non Agricultural laborer– 4.3%	Land ownership
	Family Helper– 0.2%	Yes– 95.3%
	Other work– 0.2%	No– 3.7%
	Work and attend educational institution– 0.9%	Don’t’ know– 1.0%
	Domestic duty– 29.0%	
	Not working, in school– 13.8%	
	Seeking work– 2.8%	
	Not able to work due to impairment– 4.8%	
	Retired– 7.1%	
	Does not want to work– 0.5%	

In Nepal, the mean age of all married women respondents was 31.97 years. Approximately 56% of the sample was illiterate and 66% had not attended school. The mean household yearly income was over half a million NPRs ($5250) where approximately 95% of the household heads indicated owning land, and all of them were primarily employed in agricultural work.

### Construct validity for the initial DSQ-27

Table 2 reports results for sensitivity, specificity, positive likelihood ratio (LHR+), negative likelihood ratio (LHR-) positive predictive value (PPV+), negative predictive value (NPV-) for the original DSQ-27 elaborated and tested in Afghanistan. The DSQ-27 correctly differentiated individuals with and without impairment. The ability of the included scales for correctly classifying (CC) disability–both true positives and true negatives–ranged from 61.1% for female children to 89.6% for male adults, with an overall average CC of 76.7%. The average positive predictive value of 92.5% shows that almost all respondents with a positive test actually had a disability. Conversely, the proportion of the sample without a disability that tested negatively is 67.2%, with a minimum of 48.1% for female children. With an overall sensitivity of 62.9% and a specificity of 93.7%, the DSQ-27 appears to be an overall robust test. Sensitivity was significantly lower than specificity for adults suggesting that adults with a disability do not differ enough from their non-disabled counterparts on the DSQ-27. An item per item exploration revealed that respondents, both children and adults, were not adequately identified by the DSQ-27 as having difficulties in activity of daily living such as bathing, getting dressed, going to the toilet or moving around. Similarly, the DSQ-27 did not capture difficulties with remembering things, understanding others and being understood by others, but also feeling of sadness, oppression and suffocation (data not shown). All these items were added in the revised version used in following settings (i.e. India and Nepal).

**Table 2:** Sensitivity and specificity of the DSQ-27 in Afghanistan.

	n (%)	True positive	False positive	True negative	False negative	CC	Sensitivity (95%CI)	Specificity (95%CI)	PLR (95%CI)	NLR (95%CI)	Condition prevalence	PPV (95%CI)	NPV (95%CI)
**Male children (5–14)**	677	161 (23.8)	15 (2.2)	294 (43.4)	207 (30.6)	67.2	43.75 (38.61–48.99)	95.15 (92.12–97.26)	9.01 (5.43–14.96)	0.59 (0.54–0.65)	54.36 (50.52–58.16)	91.48 (86.33–95.15)	58.68 (54.23–63.03)
**Female children (5–14)**	507	132 (26)	5 (1.0)	178 (35.1)	192 (37.9)	61.1	40.74(35.34–46.31)	97.27 (93.73–99.10)	14.91 (6.22–35.75)	0.61 (0.55–0.67)	63.91 (59.55–68.09)	96.35 (91.68–98.79)	48.11 (42.91–53.33)
**Male adults (> = 15)**	865	375 (43.4)	43 (5.0)	400 (46.2)	47 (5.4)	89.6	88.86 (85.47–91.70)	90.29 (87.15–92.89)	9.15 (6.88–12.19)	0.12 (0.09–0.16)	48.79 (45.41–52.17)	89.71 (86.39–92.45)	89.49 (86.26–92.17)
**Female adults (> = 15)**	647	267 (41.3)	13 (2.0)	261 (40.3)	106 (16.4)	81.6	71.58 (66.71–76.11)	95.26 (92.02–97.45)	15.09 (8.84–25.75)	0.30 (0.25–0.35)	57.65 (53.74–61.49)	95.36 (92.19–97.50)	71.12 (66.19–75.70)
**Overall**	2696	935(34.7)	76 (2.8)	1133 (42.0)	552 (20.5)	76.7	62.88 (60.37–65.34)	93.71 (92.19–95.02)	10 (8.02–12.48)	0.4 (0.37–0.42)	55.16 (53.26–57.05)	92.48 (90.68–94.03)	67.24 (64.94–69.48)

Notes. CC = Correct classification; SN = Sensitivity; SP = PPV = Positive predictive value; Negative predictive value; LR+ = Positive likelihood ratio; LR- = Negative likelihood ratio.

### Internal consistency

Internal consistency as indicated by a raw/standardized Cronbach’s Alpha, was 0.83/0.82 for India and 0.76/0.78 for Nepal. Both alphas were very close to each other in magnitude and above the established level 0.70 for an acceptable scale [16]. The standardized Cronbach’s Alpha was used to assess internal consistency for each domain in India/Nepal: Mobility/Physical (0.54/0.54), Sensory (0.42/0.37), Intellectual/Developmental Delay (0.68/0.60), Behavioral Patterns (0.76/0.65), Mood/Emotions functioning difficulties (0.67/0.46), and Neurological difficulties (0.68/0.64). In the India survey, one out of the six domain specific alphas are above the acceptable level of 0.70; but none in the Nepal survey. In the India survey, question #5 in the Mobility/Physical domain was excluded from the analysis because of a lack of variance (all responses indicated no). In both surveys, question #32 in the mood/emotions domain was excluded from analysis, as all respondents were older than 15 years of age. The exclusion of these questions may have contributed to a lower alpha in their respective domain and survey.

### Exploratory factor analyses

The Kaiser-Meyer-Olkin measure of sampling was well above the accepted cutoff of 0.40 for India (0.82) and Nepal (0.82). The criteria for Bartlett’s test of sphericity were also met for both India (< .001) and Nepal (< .001) [17]. There were 9 factors that explained 40% and 32% of the variance respectively in the India and the Nepal survey (S1 Table). In the India survey, the first factor (7 items) was best characterized as changes in mood or behaviors. The second factor (2 items) consisted of epilepsy symptoms while the third factor (3 items) encompassed difficulties in communicating. The fourth factor (2 items) was characterized as behavior and slow development and the fifth factor (4 items) consisted of difficulties with physical functioning. The sixth factor (3 items) was characterized by psychosomatic symptoms (e.g. hallucinations); the seventh factor (2 items) centered on sensory impairments while the eighth factor (2 items) encompassed developmental delay. The ninth factor (2 items) focused on mood regulation. There were 8 items that did not load on the ten factors because of absolute values <0.4. In the Nepal survey, there were similar factor loadings (S2 Table). The first factor (5 items) characterized difficulty with communication, both expression and comprehension. The second factor (6 items) encompassed mood and behavior problems. The third factor (3 items) characterized epilepsy symptoms while the fourth factor (2 items) characterized psychosomatic symptoms. The fifth factor (2 items) centered on developmental delay and the sixth encompassed mobility impairment. Both the seventh (2 items) and ninth (1 item) factors were characterized as disability/limitations while the eight factor did not have any item, which loaded above 0.4. There were 11 items that did not load on the 9 factors due to a low absolute value. Since factors 9 and 10 in the India survey and factors 8 and 9 in the Nepal survey had less than two items loading, no meaningful interpretation can be attribute to them [18]. Based on the EFA, the order and number of items in the DSQ-34 does not accurately match the a priori domains assigned during the development and refinement stages. However, there are several strong overlapping factors across both surveys that do match the putative domains assigned. These include: changes in mood/emotion and behaviors, difficulty with communication (receptive and expressive), psychosomatic symptoms (e.g. hallucinations), developmental delays, neurological symptoms of epilepsy and physical disabilities that impact mobility. This cadre of six domains contains the majority of the items in the DSQ-34 and matches the a priori titled domains.

### Reliability

The test-retest reliability for India (n = 65) and Nepal (n = 53) were established with participants who were tested two weeks apart. The intraclass correlation for India and Nepal respectively reached ICC = 0.75 (*p* < .001) and ICC = 0.77 (*p* < .001), both above the acceptable level of 0.70 [16].

Inter-rater reliability for India (n = 90) and Nepal (n = 50) were established with participants being tested the same day by different raters. The weighted kappa statistics were 0.77 for India and 0.79 for Nepal—above 0.70—reaching good strength of agreement [16]. These results were further confirmed by calculating Bland-Altman plots for India (5.8%) and Nepal (6.0%) showing low percentage of points outside of the limits of agreements using 95% confidence interval [19–21].

### Responsiveness

The SEM/MDC for India (0.80/2.22) and Nepal (0.96/2.66) were very small (<1) and were close to each other indicating a smaller amount of measurement error. The MDC based on a 95% confidence interval was also small and indicates the minimum amount of change in a participant’s score that is required to confirm the change is not a result of measurement error.

Established cut-offs indicate that ≤15% of a sample’s response are needed to demonstrate no floor effects or ceiling effects [16]. Floor effects indicated that 84.4% of the India respondents and 68.3% of the Nepal respondents had the lowest score possible (0) on the DSQ-34. While a majority of respondents had the lowest score (0), this is not indicative of a floor effect. The purpose of the DSQ-34 is to assess activity limitations and functioning difficulties; the results indicate no such self-reported limitations and difficulties in both populations. Ceiling effects were 0% for both India and Nepal indicating no ceiling effects.

We computed a series of *t*-tests to examine group differences between respondents with activity limitations and functioning difficulties (cases, n = 2551) and non-disabled respondents in India (controls, n = 2738) among the domains and the overall survey. There was no significant difference between cases and controls in gender (*p* = .214) and in monthly income (*p* = .149). On average, cases were significantly slightly older than controls (*p* = < .001), did not have education beyond high school (*p* = < .001) and were more often unmarried (*p* = < .001). There was a statistically significant difference (at least *p* < .05) in the raw total score and all domains (see Table 3). On average, all means and standard deviations were higher for cases when compared to controls, suggesting the DSQ-34’s ability to discriminate between persons who have reported activity limitations or functioning difficulties compared to non-disabled controls.

**Table 3:** Group differences on domains and overall score in India.

Domains					95% CI	
		Mean	SD	*p*	Upper	Lower
Raw total	Case	0.89	2.55	< .001	0.591	0.798
	Control	0.19	1.00			
Mobility/Physical	Case	0.10	0.49	0.021	0.004	0.054
	Control	0.07	0.44			
Sensory	Case	0.04	0.27	0.018	0.003	0.030
	Control	0.03	0.23			
Intellectual/Developmental Delay	Case	0.20	0.81	< .001	0.137	0.203
	Control	0.03	0.34			
Behavioral Patterns	Case	0.36	1.26	< .001	0.275	0.373
	Control	0.04	0.34			
Moods/Emotion	Case	0.17	0.72	< .001	0.117	0.174
	Control	0.02	0.25			
Neurological	Case	0.01	0.17	0.003	0.003	0.016
	Control	0.00	0.04			

## Discussion

We evaluated the measurement properties of a screening questionnaire developed in Afghanistan and validated in India and Nepal to identify various domains of activity limitations and functioning difficulties. To our knowledge, the current absence of a gold standard measure of disability available for LMICs did not allow us to test convergent validity with the DSQ-34. In Nepal and India, the DSQ-34’s strong psychometric properties indicate that it effectively discriminates between persons with activity limitations and functioning difficulties and the rest of the population.

### Accuracy, completeness and content validity

The DSQ-27 provided good accuracy in identifying persons with activity limitations and functioning difficulties associated with impairment. The subgroup measures by gender and age had good PR+ values in order to differentiate respondents with and without activity limitations and functioning difficulties. Missing items were added to the revised version tested in India and Nepal to improve accuracy.

The DSQ-34 provides information on a broader range of activity limitations and functioning difficulties than the WG short questionnaire, the WHODAS-2 36-item scale or the section 2 “assessment of functioning” 17-item of the RAD [22, 23]. More specifically, both the WG and RAD do not contain items on behavioral difficulties and neurological disorders. The ability to identify various mental impairments besides psychological distress identified by the RAD questionnaire is of paramount importance for designing programs and policies for persons with disabilities. Behavioral and neurological disorders are particularly stigmatized in various cultural contexts of LMICs and as a result, these individuals are extremely vulnerable to poverty and face the risk of further exclusion from development efforts [24–27].

The DSQ-34’s ability to be used in different cultural contexts was evident in the English, Hindi and Nepali translations. Respondents did not face difficulties in understanding or interpreting the questions. This is reflected in the low level of missing responses: below 0.5% in both India and Nepal. Since respondents were randomly selected and screened at the same time and the same place, it is very unlikely that selection bias influenced our study results [28]. There was a problem of recall bias among adult respondents in the case of being “late to talk and to walk”, especially in Nepal. However, this did not lead to an underestimation of learning difficulties because recall bias was only an issue for minor delays (less than 2 years) and not for delays of over two years, which would indicate a significant learning impairment. Respondents with slight delays in achieving the milestones of talking or walking (by less than a year) had difficulty recalling it; therefore small delay were not always reported.

### Consistency, reliability and agreement

The DSQ-34 demonstrated good internal consistency, acceptable level of ICC, good test-retest, and inter-rater reliability and good level of agreement. Reliability was confirmed by good responsiveness measurement. This indicates that the DSQ-34 can discriminate adequately between individuals with and without disability and reflect change in condition over time.

### Identification of persons with disabilities

A reliable disability-screening questionnaire should be sensitive and specific in identifying only persons with activity limitations and functioning difficulties [29]. The DSQ-34 demonstrated adequate sensitivity and specificity. More importantly, it demonstrated the ability to identify specific difficulties associated across subdomains. The complexity of disability as a phenomenon reflects the interdependent relationship between the person’s impairment, ability to function independently and the environment. For example, individuals with mobility limitations might develop anxiety and distress due to participation restrictions linked to lack of accessibility as well as to the collective attitudes [30]. Similarly, persons with mental illness might develop low self-esteem linked to prejudice and discrimination they face in their community resulting in mood and affect disorders and overall worsening of mental health [31].

### Screening for disability as a tool to be used in development and epidemiological studies

Contrary to the MDS, the DSQ-34 does not seek to collect information about all dimensions of disabilities allowing for comparison across cultural contexts. The DSQ-34 is a measure of activity limitations and functioning difficulties in everyday life for persons living in LMIC’s where prevalence of such limitations/difficulties is estimated to be higher than in HIC [8]. The DSQ-34 does not aim to evaluate other important principles defined by the UNCRPD in its article 3, in particular non-discrimination of persons with disabilities, participation in society, respect for difference, equality of opportunity, accessibility of services, gender equality, respect of evolving capacities of children with disabilities [32]. The DSQ-34 can be used with other instruments tailored to specific contexts and/or topics to examine factors that influence disability and assess the extent to which persons with disabilities are treated equally in their community [2]. Following Sen (1982), we argue that equality of capabilities is critical to flourishing and should be the main object of inclusive human development programs and policies [13, 33, 34]. This is paramount as the growing body of literature shows that persons with disabilities in LMIC are more often poorer than non disabled people [35, 36], have less access to education [37], employment [38] and healthcare services [39, 40], and face disempowerment and stigma [41, 42]. The fact that persons with disabilities in the current studies were found to be less educated, more often unmarried, and poorer than non-disabled people reflects the inequality of opportunities and difficult circumstances.

### Limitations

The DSQ-34 does not give a conclusive, discriminative diagnostic of impairment but only the identification of overall activity limitations and functioning difficulties. More refined and specific questionnaires are required to explore a specific condition that may be associated with a disability. Additionally, the DSQ-34 establishes prevalence of impairment and should work as an adjunct with other tools assessing aspects of disability such as participation restrictions, development concerns and individual aspirations. We did not use traditional cognitive testing to assess clarity and relevance of each item or to identify the mental process the respondent followed to interpret the question and to formulate a response [43, 44]. Yet, our team engaged informally with respondents to discuss their understanding of the question, looking at how the activity limitation or the functioning difficulty translated in their daily life when they mentioned they were facing one in response to any of the items.

Our study shows the potential of using a simple, standardized screening questionnaire to identify persons with disabilities. We anticipate that the DSQ-34 will facilitate the collection of data that is currently lacking on disabled populations. The DSQ-34 can be included in larger epidemiological studies without undertaking large-scale, expensive disability-specific studies, requiring significant resources to gather a representative sample. The need to build such an evidence-base is clearly addressed in Article 31 of the UNCRPD, which stipulates that the collection of information and, particularly, statistical data required for the design and implementation of appropriate policies, is the responsibility of State parties. The DSQ-34 is a strong contribution towards monitoring the UNCRPD and progress towards the Sustainable Development Goals (SDGs) for people with disabilities as part of populations-based SDGs efforts.

## Supporting Information

S1 File Disability Screening Tool 34 items. (DOCX)

S2 File Details about Methods. (DOCX)

S1 Table Exploratory factor analysis using PAF and varimax rotation for India survey. (DOCX)

S2 Table Exploratory factor analysis using PAF and varimax rotation for Nepal survey. (DOCX)

## References

[1] Eide AH, Jelsma J, Loeb M, Maart S, Toni MKA. Exploring ICF components in a survey among Xhosa speakers in Eastern & Western Cape, South Africa. Disability and Rehabilitation. 2008;30(11):819–29. 10.1080/09638280701390998 . 17852254

[2] Bakhshi P, Trani JF, Rolland C, Handicap I, Afghanistan. Conducting Surveys on Disability: A Comprehensive Toolkit. Lyon: Handicap International Afghanistan Programme; 2006.

[3] Altman BM. Definitions of disability and their operationalization, and measurement in survey data: an update In: Altman BM, Barnartt BM, editors. Exploring theories and expanding methodologies: where we are and where we need to go. 2 Oxford: Elsevier Jai; 2001 p. 77–100.

[4] Marella M, Busija L, Amirul Islam FM, Devine A, Fotis K, Baker SM, et al Field-testing of the rapid assessment of disability questionnaire. BMC Public Health. 2014;14(1). 10.1186/1471-2458-14-900 PMC4246465 25179800

[5] Hosseinpoor AR, Williams JAS, Gautam J, Posarac A, Officer A, Verdes E, et al Socioeconomic inequality in disability among adults: A multicountry study using the world health survey. American Journal of Public Health. 2013;103(7):1278–86. 10.2105/AJPH.2012.301115 23678901 PMC3682610

[6] Mitra S, Sambamoorthi U. Disability prevalence among adults: Estimates for 54 countries and progress toward a global estimate. Disability and Rehabilitation. 2014;36(11):940–7. 10.3109/09638288.2013.825333 23962193

[7] Mont D. Measuring health and disability. Lancet. 2007;369(9573):1658–63. 17499607 10.1016/S0140-6736(07)60752-1

[8] World Health Organization, World Bank. World report on disability Geneva: World Health Organization, 2011.

[9] Madans JH, Loeb M. Methods to improve international comparability of census and survey measures of disability. Disability and Rehabilitation. 2013;35(13):1070–3. 10.3109/09638288.2012.720353 23020151

[10] Mont D. Measuring disability prevalence Washington, DC: World Bank, 2007.

[11] Mitra S. The capability approach and disability. Journal of Disability Policy Studies. 2006;16(4):236–47.

[12] Trani JF, Bakhshi P. Challenges for assessing disability prevalence: The case of Afghanistan. ALTER European Journal of Disability Research. 2008;2:44–64.

[13] Dubois JL, Trani JF. Extending the capability paradigm to address the complexity of disability. ALTER European Journal of Disability Research. 2009;3(3):192–218.

[14] Sabariego C, Oberhauser C, Posarac A, Bickenbach J, Kostanjsek N, Chatterji S, et al Measuring disability: Comparing the impact of two data collection approaches on disability rates. International Journal of Environmental Research and Public Health. 2015;12(9):10329–51. 10.3390/ijerph120910329 26308039 PMC4586614

[15] World Health Organization, World Bank. WHO MDS Survey Manual: The WHO Model Disability Survey Geneva: World Health Organization, 2014.

[16] Terwee CB, Bot SDM, de Boer MR, van der Windt DAWM, Knol DL, Dekker J, et al Quality criteria were proposed for measurement properties of health status questionnaires. Journal of Clinical Epidemiology. 2007;60(1):34–42. 17161752 10.1016/j.jclinepi.2006.03.012

[17] Williams B, Brown T, Onsman A. Exploratory factor analysis: A five-step guide for novices. Australasian Journal of Paramedicine. 2012;8(3):1–13.

[18] Henson RK, Roberts JK. Use of exploratory factor analysis in published research: Common errors and some comment on improved practice. Educational and Psychological Measurement. 2006;66(3):393–416.

[19] altman DG, Bland JM. Measurement in medicine: the analysis of method comparison studies. The Statistician. 1983;32:307–17.

[20] Bland JM, Altman DG. Measurement in medicine: The analysis of method comparison studies. Statistician. 1983;32:307–17.

[21] Bland JM, Altman DG. Statistical methods for assessing agreement between two methods of clinical measurement. Lancet. 1986;1(8476):307–10. 2868172

[22] Lira Huq N, Edmonds TJ, Baker S, Busjia L, Devine A, Fotis K, et al The rapid assessment of disability—Informing the development of an instrument to measure the effectiveness of disability inclusive development through a qualitative study in Bangladesh. Asia Pacific Disability Rehabilitation Journal. 2013;24(3):37–60.

[23] Madans JH, Loeb ME, Altman BM. Measuring disability and monitoring the UN Convention on the Rights of Persons with Disabilities: the work of the Washington Group on Disability Statistics. BMC Public Health. 2011;11. doi: S4 10.1186/1471-2458-11-s4-s4 . PMC3104217 21624190

[24] Bhalla D, Chea K, Hun C, Vannareth M, Huc P, Chan S, et al Population-Based Study of Epilepsy in Cambodia Associated Factors, Measures of Impact, Stigma, Quality of Life, Knowledge-Attitude-Practice, and Treatment Gap. Plos One. 2012;7(10). 10.1371/journal.pone.0046296 PMC3471879 23077505

[25] Lim KS, Tan CT. Epilepsy stigma in Asia: The meaning and impact of stigma. Neurology Asia. 2014;19(1):1–10.

[26] Lim YJ, Chan SY, Ko Y. Stigma and health-related quality of life in Asian adults with epilepsy. Epilepsy Research. 2009;87(2–3):107–19. 10.1016/j.eplepsyres.2009.08.014 19782536

[27] Alonso J, Buron A, Bruffaerts R, He Y, Posada-Villa J, Lepine JP, et al Association of perceived stigma and mood and anxiety disorders: results from the World Mental Health Surveys. Acta Psychiatrica Scandinavica. 2008;118(4):305–14. 10.1111/j.1600-0447.2008.01241.x 18754833 PMC3040096

[28] Savitz DA. Interpreting Epidemiologic Evidence: Strategy for Study Design and Analysis: Oxford, UK: Oxford University Press; 2003.

[29] Goujon N, Devine A, Baker SM, Sprunt B, Edmonds TJ, Booth JK, et al A comparative review of measurement instruments to inform and evaluate effectiveness of disability inclusive development. Disability and Rehabilitation. 2014;36(10):804–12. 10.3109/09638288.2013.821178 23930646

[30] Velema JP, Ebenso B, Fuzikawa PL. Evidence for the effectiveness of rehabilitation-in-the-community programmes. Leprosy Review. 2008;79(1):65–82. 18540238

[31] Link BG, Struening EL, Neese-Todd S, Asmussen S, Phelan JC. The consequences of stigma for the self-esteem of people with mental illnesses. Psychiatric Services. 2001;52(12):1621–6. 10.1176/appi.ps.52.12.1621 . 11726753

[32] Convention on the rights of persons with disabilities, (2006). 10.1515/9783110208856.203 18348362

[33] Sen AK. Equality of What? Choice, Welfare and Measurement. 353–369. Oxford: Blackwell; 1982.

[34] Trani JF, Bakhshi P, Bellanca N, Biggeri M, Marchetta F. Disabilities through the Capability Approach lens: Implications for public policies. Alter. 2011;5(3):143–57.

[35] Groce N, kett M., Lang R., Trani JF. Disability and poverty: the need for a more nuanced understanding of implications for development policy and practice. Third World Quarterly. 2011;32(8):1493–513.

[36] Mitra S, Posarac A, Vick B. Disability and Poverty in Developing Countries: A Multidimensional Study. World Development. 2013;41(1):1–18.

[37] Filmer D. Disability, poverty and schooling in developing countries: results from 14 household surveys. The World Bank Economic Review. 2008;22:141–63.

[38] Mizunoya S, Mitra S. Is There a Disability Gap in Employment Rates in Developing Countries? World Development. 2013;42(1):28–43.

[39] Trani J-F, Browne J, Kett M, Bah O, Morlai T, Bailey N, et al Access to health care, reproductive health and disability: A large scale survey in Sierra Leone. Social Science & Medicine. 2011;73(10):1477–89. 10.1016/j.socscimed.2011.08.040 . 22014873

[40] Trani J-F, Bakhshi P, Noor AA, Lopez D, Mashkoor A. Poverty, vulnerability, and provision of healthcare in Afghanistan. Social Science & Medicine. 2010;70(11):1745–55. 10.1016/j.socscimed.2010.02.007 . 20359809

[41] Kassah AK. Community-based rehabilitation and stigma management by physically disabled people in Ghana. Disability & Rehabilitation. 1998;20(2):66–73. 9494040 10.3109/09638289809166056

[42] Parnes P, Cameron D, Christie N, Cockburn L, Hashemi G, Yoshida K. Disability in low-income countries: issues and implications. Disability & Rehabilitation. 2009;31(14):1170–80. 19802932 10.1080/09638280902773778

[43] DeMaio T, Rothgeb J. Cognitive interviewing techniques: in the lab and in the field In: Schwarz N, Sudman S, editors. Answering Questions Methodology for Determining Cognitive and Communicative Processes in Survey Research. San Francsico:: Jossey-Bass, Ine; 1996 p. 177–96.

[44] Tourangeau R, Rips LJ, Rasinski K. The psychology of survey response: Cambridge University Press; 2000.

