# Supplementary material for: Development and Validation of the 34-Item Disability Screening Questionnaire (DSQ-34) for Use in Low and Middle Income Countries Epidemiological and Development Surveys
Source: PLoS One. 2015 Dec 2;10(12):e0143610. doi: 10.1371/journal.pone.0143610 (PMC4667846; doi:10.1371/journal.pone.0143610)
Supplement: S1 File — (DOCX) [file pone.0143610.s001.docx]

**Disability Screening Tool 34 items**

1. Do you lack part of one or more limb?

4 = YES, I lack two or more limbs or part of several limb

3 = YES, I lack one limb

2 = YES, I lack part of one limb

1 = NO, I do not lack any limb at all

1. Are you partially or totally paralysed?

4 = YES, I am totally paralysed, my all body is paralysed,

3 = YES, I am half paralysed, only my upper/lower part of the body is paralysed

2 = YES, but less than half of my body is paralysed, one leg or one arm

1 = NO, I am not paralysed at all

1. Are you unable to move part or entire body?

4 = YES, I am totally unable to move my entire body

3 = YES, I have a lot of difficulty to move part or my entire body

2 = YES, I have some difficulty to move part or my entire body: I move slowly

1 = NO, I have no difficulty to move my body at all

1. Do you have any difficulties walking/moving around or climbing steps?

4 = YES, I am totally unable to walk or climb steps

3 = YES, I have a lot of difficulty to walk or climb steps

2 = YES, I have some difficulty to walk or climb steps: I walk at a slow pace and take more time than anyone to climb steps

1 = NO, I have no difficulty at all to walk or climb steps

1. Do you have a part of the body that looks different from the other persons of the family?

2 = YES, I have part of the body that looks different

1 = NO, I have no part of the body that looks different

1. Do you have difficulty or need assistance taking care of yourself, such as bathing, getting dressed, or going to the toilet?

4 = YES, constantly, I always need assistance to take care of myself

3 = YES, I often need assistance to take care of myself

2 = YES, I sometimes need assistance to take care of myself

1 = NO, I never need assistance to take care of myself

1. Do you have any difficulties seeing even if wearing glasses?

4 = YES, I cannot see at all

3 = YES, I have a lot of difficulty to see, and I need glasses all the time

2 = YES, I have some difficulty to see, I need glasses sometime (for reading, for seeing near things or for seeing far away…)

1 = NO I have no difficulty to see at all

1. Do you have any difficulties hearing, even if using a hearing aid?

4 = YES, I cannot hear at all

3 = YES, I have a lot of difficulty to hear, I need a hearing aid

2 = YES, I have some difficulty to hear but I don’t need an hearing aid

1 = NO, I have no difficulty to hear at all

1. Are you unable to talk, or have you difficulties pronouncing words?

4 = YES, I cannot talk at all

3 = YES, I have a lot of difficulty to talk, pronounce words

2 = YES, I have some difficulty to talk, pronounce words

1 = NO, I have no difficulty to talk at all

1. Did anybody in your mother’s home tell you that you were later than other children beginning to walk? (above 4 years old)

4 = YES, I was a lot later, several years (more than two) later than other children beginning to walk

3 = YES, I was more than a year later than other children beginning to walk

2 = YES, I was slightly late, about one year or less later, than other children beginning to walk

1 = NO, not later than others beginning to walk

1. Did anybody in your mother’s home tell you that you were later than other children beginning to talk? (above 4 years old)

4 = YES, I was a lot later, more than 5 years later than other children beginning to talk

3 = YES, I was rather late, between 2 and 5 years later than other children beginning to talk

2 = YES, slightly late about 1 year or less later than other children beginning to talk

1 = NO, I was not later than other children beginning to talk

1. Do you have more difficulty or are you slower than others in learning things and do you need to be constantly encouraged to do things? How often?

4 = YES, I cannot learn at all or with very high difficulty

3 = YES, I have a lot of difficulty to learn and to do things

2 = YES, I have some difficulty to learn and to do things

1 = NO, I have no difficulty to learn and to do things at all

1. Do people think or say that you have difficulty acting/behaving your own age and like behaving much younger than your own age? How often they think you do?

4 = YES, I constantly/ always have difficulty acting/behaving my own age and I always like to behave younger

3 = YES, I often/ many times have difficulty acting/behaving my own age and I often like to behave younger

2 = YES, sometimes/few times have difficulty acting/behaving my own age and I sometimes like to behave younger

1 = NO, I never have difficulty acting/behaving my own age, I don’t like behaving younger

1. Do you have difficulty in generally understanding what people are telling you? How often?

4 = YES, constantly /always (I have constantly /always difficulty in generally understanding what people are telling me)

3 = YES, often (I have often difficulty in generally understanding what people are telling me)

2 = YES, sometimes (I have sometimes difficulty in generally understanding what people are telling me)

1 = NO, never (I have no difficulty in generally understanding what people are telling me)

1. Do you have difficulty to generally make yourself understood by others? How often?

4 = YES, constantly /always (I have constantly /always difficulty generally to make myself understood by others)

3 = YES, often (I have often difficulty generally to make myself understood by others)

2 = YES, sometimes (I have sometimes difficulty generally to make myself understood by others)

1 = NO, never (I have never difficulty generally to make myself understood by others)

1. Do you have difficulty concentrating or remembering things? How often?

4 = YES, constantly /always (I constantly /always have difficulty concentrating or remembering things)

3 = YES, often (I have often difficulty concentrating or remembering things)

2 = YES, sometimes (I have sometimes difficulty concentrating or remembering things)

1 = NO, never (I have never difficulty concentrating or remembering things)

1. Do people think or say that you behave in a strange manner? How often they think you behave as such?

4 = YES, constantly /always (they think I constantly /always behave in a strange manner)

3 = YES, often (they think I often behave in a strange manner)

2 = YES, sometimes (they think I sometimes behave in a strange manner)

1 = NO, never (they think I never behave in a strange manner)

1. Do you think that someone wants to hurt you, to be nasty to you or has done something bad to you and when you share this with other people nobody believes you, or people laugh at you or they become angry? How often?

4 = YES, constantly /always (I constantly /always think that someone wants to hurt me, people don’t believe me)

3 = YES, often (I often think that someone wants to hurt me, people don’t believe me)

2 = YES, sometimes (I sometimes think that someone wants to hurt me, people don’t believe me)

1 = NO, never (I never think that someone wants to hurt me, people don’t believe me)

1. Do you see or hear things and when you share this with other people they do not believe you? How often?

4 = YES, constantly /always (I constantly /always see or hear things and when I share this with other people they don’t believe me)

3 = YES, often (often see or hear things and when I share this with other people they don’t believe me)

2 = YES, sometimes (sometimes see or hear things and when I share this with other people they don’t believe me)

1 = NO, never (I never see or hear things see or hear things)

1. Do you talk aloud when you are alone? How often?

4 = YES, constantly /always (I constantly /always talk to myself aloud when I am alone)

3 = YES, often (I often talk to myself aloud when I am alone)

2 = YES, sometimes (I sometimes talk to myself aloud when I am alone)

1 = NO, never (I never talk to myself aloud when I am alone)

1. Do you feel good making the same gestures over and over again like rocking (show how), biting your arm, swinging your head? How often?

4 = YES, constantly /always (I always feel good making the same gestures over and over again)

3 = YES, often (I often feel good making the same gestures over and over again)

2 = YES, sometimes (I sometimes feel good making the same gestures over and over again)

1 = NO, never (I never feel good making the same gestures over and over again)

1. Are you afraid when you are touched or hear a noise that you do not like? How often this happens?

4 = YES, constantly /always (I am always afraid when someone touches me or if I hear a noise that I do not like)

3 = YES, often (I am often afraid when someone touches me or if I hear a noise that I do not like)

2 = YES, sometimes (I am sometimes afraid when someone touches me or if I hear a noise that I do not like)

1 = NO, never (I am never afraid when someone touches me or if I hear a noise that I do not like)

1. Do you feel better when you hit your head against the wall, or when you pull your hair when you are upset, sad or angry? How often do you do it?

4 = YES, constantly /always (I always hit my head against the wall, or pull my hair when I am upset, sad or angry)

3 = YES, often (I often hit my head against the wall, or pull my hair when I am upset, sad or angry)

2 = YES, sometimes (I sometimes hit my head against the wall, or pull my hair when I am upset, sad or angry)

1 = NO, never (I never hit my head against the wall, or pull my hair when I am upset, sad or angry)

1. Does it make you upset, sad or angry when things are not the same or when someone changes things around you? How often does it make you upset, sad or angry?

4 = YES, constantly /always (it make me always upset, sad or angry when things are not the same or when someone changes things around me)

3 = YES, often (it make me often upset, sad or angry when things are not the same or when someone changes things around me)

2 = YES, sometimes (it make me sometimes upset, sad or angry when things are not the same or when someone changes things around me)

1 = NO, never (it never make me upset, sad or angry when things are not the same or when someone changes things around me)

1. Are you happy being alone or do you prefer to be with family or other people you like? How often do you prefer staying alone?

4 = YES, constantly /always (I always prefer to be alone than with family or other people I like)

3 = YES, often (I often prefer to be alone than with family or other people I like)

2 = YES, sometimes (I sometimes prefer to be alone than with family or other people I like)

1 = NO, never (I never prefer to be alone than with family or other people I like)

1. Are you angry with other people and want to find with them and they don’t understand why you are angry? How often are you angry with people?

4 = YES, constantly /always (I am always angry with other people)

3 = YES, often (I am often angry with other people)

2 = YES, sometimes (I am sometimes angry with other people)

1 = NO, never (I am never angry with other people)

1. Do you feel happy, and then immediately sad, or happy and then immediately angry (one moment you are happy and one moment you are sad or angry)? How often you feel like that?

4 = YES, constantly /always (I feel always happy and then sad or angry immediately)

3 = YES, often (I feel often happy and then sad or angry immediately)

2 = YES, sometimes (I feel sometimes happy and then sad or angry immediately)

1 = NO, never (I never feel happy and then sad or angry immediately)

1. Do you want to or like moving around and hate keeping still or sit in one place for long? How often does this happen

4 = YES, constantly /always (I always want to move around and hate keeping still or sit in one place for long)

3 = YES, often (I often want to move around and hate keeping still or sit in one place for long)

2 = YES, sometimes (I sometimes want to move around and hate keeping still or sit in one place for long)

1 = NO, never (I never want to move around and never hate keeping still or sit in one place for long)

1. Do you **not** feel worried or sad when someone close to you is hurt, upset or sad? How often does this happen?

4 = YES, constantly /always (I always feel **not** worried or sad when someone close to me is hurt, upset or sad)

3 = YES, often (I often feel **not** worried or sad when someone close to me is hurt, upset or sad)

2 = YES, sometimes (I sometimes feel **not** worried or sad when someone close to me is hurt, upset or sad)

1 = NO, never (I never feel **not** worried or sad when someone close to me is hurt, upset or sad)

1. Do you **not** notice when someone is speaking to you, or are you not aware of the presence of others? How often does this happen?

4 = YES, constantly /always (I always **not** notice when someone is speaking to me)

3 = YES, often (I often **not** notice when someone is speaking to me)

2 = YES, sometimes (I sometimes **not** notice when someone is speaking to me)

1 = NO, never (I never **not** notice when someone is speaking to me)

1. Do you feel afraid for no reason or feel easily scared? How often does this happen?

4 = YES, constantly /always (I always feel afraid for no reason)

3 = YES, often (I often feel afraid for no reason)

2 = YES, sometimes (I sometimes feel afraid for no reason)

1 = NO, never (I never feel afraid for no reason)

1. Have you fainted in the past 3 months? How often did this happen?

4 = YES, constantly /always (I fainted more than 40 times in the last 3 months)

3 = YES, often (I fainted between 5 and 39 times in the last 3 months)

2 = YES, sometimes (I fainted 1 or 2 times in the last 3 months)

1 = NO, never (I never fainted)

1. Did you experience sudden jerking of (or parts of) the body with loss of consciousness during the last 3 months? How often did this happen?

4 = YES, constantly /always (I experienced sudden jerking more than 40 times in the last 3 months)

3 = YES, often (I experienced sudden jerking 5 to 40 times in the last 3 months)

2 = YES, sometimes (I experienced sudden jerking 1 or 2 times in the last 3 months)

1 = NO, never (I experienced sudden jerking in the last 3 months)

1. Did you bite your tongue or froth at the mouth on occasions in last 3 months? How often did this happen?

4 = YES, constantly /always (I bit my tongue or froth at the mouth more than 40 times in the last 3 months)

3 = YES, often (I bit my tongue or froth at the mouth 5 to 40 times in the last 3 months)

2 = YES, sometimes (I bit my tongue or froth at the mouth 1 or 2 times in the last 3 months)

1 = NO, never (I bit my tongue or froth at the mouth)
