# Supplementary material for: Development and Validation of the 34-Item Disability Screening Questionnaire (DSQ-34) for Use in Low and Middle Income Countries Epidemiological and Development Surveys
Source: PLoS One. 2015 Dec 2;10(12):e0143610. doi: 10.1371/journal.pone.0143610 (PMC4667846; doi:10.1371/journal.pone.0143610)
Supplement: S2 File — (DOCX) [file pone.0143610.s002.docx]

**Appendix**

***Details about Methods***

*Content of screening questionnaire*

Building on an initial 27-item version (DSQ-27), the DSQ-34 version of the screening tool was designed to capture various concepts associated with disabilities in line with the ICF. The instrument is composed of 34 questions organized in six domains by splitting the first domain of the DSQ-27 (mobility and sensory difficulties) in two for a better understanding. The first domain, Mobility/Physical, is composed of 6 questions assessing basic activity limitations such as walking, moving around and taking care of oneself adapted respectively from the chapter 4 “mobility” and the chapter 5 “self-care” of the part d “activities and participation” of the ICF. This domain also assesses missing limbs (part s chapter 7), paralysis (part b chapter 7) and pain (part b chapter 2), and differences in body appearance such as disfigurement or exceptionally small stature (various). The second domain, Sensory, includes 3 questions aiming towards detecting difficulty in seeing, hearing and talking (part b chapter 2). The third domain, Intellectual/Developmental Delay, refers to intellectual development disorders and has 7 questions about delays in child development milestones (walking and talking) and difficulties in learning (part d chapter 1), understanding (part d chapter 3), remembering (part b chapter 4) and concentrating. The fourth domain, Behavioral Patterns, comprises 10 questions about behavioral disorders and more specifically about delusions, hallucinations, abnormal motor behavior, paranoid personality disorders (all from part b chapter 1). The fifth domain, Mood/Emotions, covers major mood and affect disorders in 6 questions: depression, anxiety, post trauma stress disorders, bipolar disorders (also from part b chapter 1). One question is for children below 15 years of age and assess whether the person has trouble getting along with other children of the same age. The sixth domain, Neurological, focuses on neurological disorders with 3 questions, specifically geared towards epilepsy and various forms of seizures (part b chapter 1). The DSQ-34 uses a 4-point Likert scale to identify the degree of severity of the activity limitations or the functioning problem (not all, sometimes, often, constantly/always) for 33 items. Question 5 in the mobility/physical domain asks about having a body part that looks different from others, is an exception and instead uses yes/no choices. The initial question “Feeling very sad/or crying without a reason” to indicate a state of depression, feeling low or sad was later modified to “Do you feel happy, and then immediately sad, or happy and then immediately angry (one moment you are happy and one moment you are sad or angry)” because respondents interpreted the question as experiencing periodic episodes of sadness, which does not constitute a functioning difficulty but instead is considered as part of everyday life.

*‘Scoring of disability*

The DSQ-34 gives both a raw total score for disability and specific scores for each of the six domains. The screen has a range of 34-134 points. Scores for each domain are divided into Mobility/Physical (22), Sensory (12), Intellectual/Developmental Delay (28), Behavioral Patterns (40), Mood/Emotions (20) and Neurological (12). The raw total score can also be transformed into a scaled total score from 0-100 using the following formula: (raw total score/134) x100. Respondents who answered “no” to all 34 questions in the disability screen were considered ‘non-disabled’. Respondents who responded “yes sometimes” to one or two items were grouped as having a ‘mild’ level of disability (minimum score of 35). Respondents were identified as being 'moderately' disabled when they reported answering at least “sometimes" to three items (score=37). They were identified as being severely disabled when they reported "Yes, often" to one to two items (37=<score=<39). Finally, they qualify as very severely disabled if they reported “constantly, always" to one item (score=38) or “often" to three items or more (score>=41) or “yes” to question 5 (score=36).

*Phases of development*

*Phase I – Initial Development stage: Afghanistan*

An initial DSQ-27 was designed by a group of experts in survey development and disability experts in Afghanistan for a national disability household survey carried out from December 2004 to July 2005 in 5250 households [[12](#_ENREF_12)]. Each question was tested with Afghans with disabilities who spoke English to ensure that they were easy to understand and that the concepts used could be translated into local languages, both Dari and Pashto. Items were selected based on five initial domains of activity limitations and functioning difficulties defined by the International Classification of Functioning, Disability and Health [[44](#_ENREF_44)]. These domains were mobility/physical and sensory difficulties; intellectual and development delay, behavioral and psychological patterns, communication and social functioning, neurological [[12](#_ENREF_12)]. The goal was to complement the questions determined by the Washington Group on Disability Statistics [[45](#_ENREF_45)]. The DSQ-27 aimed to better capture specific as well as socially stigmatized impairments across a larger number of questions with a yes or no response.

Translation in Dari and Pashto were conducted by a team of medical doctors from the Ministry of Public Health in Kabul. Back translations from Dari and Pashto to English were conducted by two professional translators from a communication company in Kabul who were blinded to the initial English version. The translations were reviewed for content validity, with particular emphasis on cultural relevance and appropriateness, by a panel composed of academics from social sciences, psychology and psychiatry background as well as international and Afghan disability experts. Discrepancies between the two documents were discussed and resolved with translators until equivalence in the three languages was reached. The Dari and Pashto versions of the questionnaire were further tested in three focus group discussions with persons with disabilities of different gender and age groups. Finally, the questionnaire was tested for content validity with 50 persons with disabilities of different gender as well as ethnicity, age, type of impairment and education background in urban and rural areas of Kabul province. The feedback obtained indicated a good understanding of questions and very few problems emerged.

In the absence of a ‘gold standard’ instrument to which our tool could be compared, a second longer screening tool was also developed based on the ICF following the same method to identify the questionnaire with the best measurement properties [[15](#_ENREF_15)]. The second tool is composed of 46 items for women, 40 items for men and 36 items for children below 15 years old. All respondents over 7 years old were interviewed about autonomy in activities of daily living (ADL (6 items), performing simple tasks outside home (5 items)[[46](#_ENREF_46)]. All respondents above 4 years old were asked about behavioral difficulties (7 items), communication difficulties (6 items), and expressing violent reactions (5 items). Adult respondents were asked about signs of anxiety (10 items). Finally, women and girls only were asked about additional indoor activities that were not relevant for men in the Afghan cultural context (6 items) [[47](#_ENREF_47)].

*Phase II –Improving sensitivity, appropriateness and relevance: Darfur*

The initial DSQ-27 elaborated in Afghanistan was extended to 34-items for a case control survey in Darfur, Sudan in November 2008. The DSQ-34 was initially tested in this context. While implementing the large-scale survey in Afghanistan, three items in need of modification were identified and items were added in the Darfur version. First, in terms of procedure, instead of asking the head of household about all the members of the household at once, we inquired about every member of the household to ensure that none was excluded. Second, we added 7 items. The original question 2 -“Is any member of your family partially or totally paralyzed/unable to move part or entire body or have problems moving around?”- was replaced by three new items: “Is (name) partially or totally paralyzed?”; “Is (name) unable to move part or entire body?”; Does (name) often have any difficulties walking, moving around or climbing steps?”. These allowed us to more adequately and comprehensively identify various mobility limitations. Three additional questions allowed us to better identify learning disabilities: “Does (name) have difficulty in generally understanding what people are telling her/him?”; Does (name) have difficulty generally to make himself/herself understood by others?; “Does (name) have difficulty concentrating or remembering things?”. We added two items to better detect difficulties relating to mood and affect: “Does (name) have rapid changes of mood, for instance feel depressed, then happy and then angry?” and “Is (name) extremely active and cannot keep still or sit in one place for long?”. These new questions increased the overall sensitivity of the questionnaire by reducing the likelihood of false negatives. Risk of false negative was identified during the fieldwork in Afghanistan. Medical doctors supervising the survey were called out for a more formal assessment when the head of household was hesitant in responding to questions about possible learning disability, mood or affect disorder of a given member of the household. Third, we replaced the yes/no response by a Likert scale. The new 34-item screening tool (DSQ-34) was tested by a team of trained male and female data collectors with respondents in West Darfur. The in depth training program was conducted during 16 days. The training content included disability theories and concepts, survey method and ethics, conducting interviews and professional principles for data collection, explanation of questions, role-plays and mock interviews, and referral mechanisms. After the classroom-based training, the team spent two days in the villages of Mindu and Borsusu (Garsila District), undertaking in-depth semi-structured interviews to test the tools. Respondents answering the screening tool were asked each question in order to identify if anyone in the household presented any of the activity limitations and functioning difficulties. They were probed with follow-up questions to explain their own understanding and interpretation of each question [[48](#_ENREF_48)]. Each interview varied between 10 minutes (for a household of two) and 30 minutes (for an household of ten members or more).

*Phase III – Establishing validity and reliability of DSQ-34 in India and Nepal*

In India and Nepal, the questionnaire was translated into Hindi and in Nepali by a bilingual group of national researchers in each country. They worked as a team to reach a near 100% accuracy on a translated scale. The Hindi and Nepali versions were then back translated into English by a different team of bilingual experts. Both teams met to discuss minor discrepancies. Both versions were pre-tested with persons with disabilities. Question 14 “Do you have more difficulty or are you slower than others learning things (change in routine, for example cooking things in a different way)” required some discussion about the type of learning and we chose the example of cooking as it was well understood by everyone, particularly by women who answered for children unable to respond. We tested various measurement properties of the DSQ-34 as reported bellow.
