# Supplementary material for: Development and Validation of the 34-Item Disability Screening Questionnaire (DSQ-34) for Use in Low and Middle Income Countries Epidemiological and Development Surveys
Source: PLoS One. 2015 Dec 2;10(12):e0143610. doi: 10.1371/journal.pone.0143610 (PMC4667846; doi:10.1371/journal.pone.0143610)
Supplement: S1 Table — (DOCX) [file pone.0143610.s003.docx]

|  | Factor | | | | | | | | |
| --- | --- | --- | --- | --- | --- | --- | --- | --- | --- |
|  | 1 | 2 | 3 | 4 | 5 | 6 | 7 | 8 | 9 |
| Q27 Mood Swings | .656 |  |  |  |  |  |  |  |  |
| Q28 Need to move around | .580 |  |  |  |  |  |  |  |  |
| Q26 Angry/want to fight | .579 |  |  |  |  |  |  |  |  |
| Q24 Upset/angry with change | .545 |  |  |  |  |  |  |  |  |
| Q25 Prefers to be alone | .486 |  |  |  |  |  |  |  |  |
| Q31 Feel afraid for no reason | .420 |  |  |  |  |  |  |  | .405 |
| Q30 Unaware when spoken to | .416 |  |  |  |  |  |  |  |  |
| Q21 Repeat same gesture continuously |  |  |  |  |  |  |  |  |  |
| Q29 Does not feel worry/sad |  |  |  |  |  |  |  |  |  |
| Q23 Feel better with self-injury |  |  |  |  |  |  |  |  |  |
| Q34 Seizures/convulsions |  | .931 |  |  |  |  |  |  |  |
| Q35 Bitten tongue/frothed at mouth |  | .743 |  |  |  |  |  |  |  |
| Q33 Fainted |  |  |  |  |  |  |  |  |  |
| Q14 Diff. understanding others |  |  | .876 |  |  |  |  |  |  |
| Q15 Diff. being understood by others |  |  | .671 |  |  |  |  |  |  |
| Q13 Acts/behave younger than age |  |  |  | .570 |  |  |  |  |  |
| Q12 Slower learning/need encouragement |  |  |  | .533 |  |  |  |  |  |
| Q16 Diff. concentrating/recall things |  |  |  |  |  |  |  |  |  |
| Q17 Behaves in strange manner |  |  |  |  |  |  |  |  |  |
| Q03 Unable to move part/entire body |  |  |  |  | .670 |  |  |  |  |
| Q04 Diff. walking around/climbing |  |  |  |  | .581 |  |  |  |  |
| Q02 Partial or total paralysis |  |  |  |  | .417 |  |  |  |  |
| Q06 Needs assistance with ADL’s |  |  |  |  | .414 |  |  |  |  |
| Q01 Lack or missing part of limb |  |  |  |  |  |  |  |  |  |
| Q07 Diff. seeing even with glasses |  |  |  |  |  |  |  |  |  |
| Q18 Think others want to hurt him/her |  |  |  |  |  | .530 |  |  |  |
| Q20 Talk aloud/conversation with self |  |  |  |  |  | .466 |  |  |  |
| Q19 See/hear things not there |  |  |  |  |  | .445 |  |  |  |
| Q09 Diff. talking/pronouncing words |  |  |  |  |  |  | .753 |  |  |
| Q08 Diff hearing even with aides |  |  |  |  |  |  | .633 |  |  |
| Q10 Later walking compared to peers |  |  |  |  |  |  |  | .746 |  |
| Q11 Later talking compared to peers |  |  |  |  |  |  |  | .689 |  |
| Q22 Afraid with touched or hear noise |  |  |  |  |  |  |  |  | .639 |

Table 3. Exploratory factor analysis using PAF and varimax rotation for India survey
