# Supplementary material for: Development and Validation of the 34-Item Disability Screening Questionnaire (DSQ-34) for Use in Low and Middle Income Countries Epidemiological and Development Surveys
Source: PLoS One. 2015 Dec 2;10(12):e0143610. doi: 10.1371/journal.pone.0143610 (PMC4667846; doi:10.1371/journal.pone.0143610)
Supplement: S2 Table — (DOCX) [file pone.0143610.s004.docx]

Table 4. Exploratory factor analysis using PAF and varimax rotation for Nepal survey

|  | Factor | | | | | | | | |
| --- | --- | --- | --- | --- | --- | --- | --- | --- | --- |
|  | 1 | 2 | 3 | 4 | 5 | 6 | 7 | 8 | 9 |
| Q15 Diff. being understood by others | .839 |  |  |  |  |  |  |  |  |
| Q14 Diff. understanding others | .771 |  |  |  |  |  |  |  |  |
| Q09 Diff. talking/pronouncing words | .755 |  |  |  |  |  |  |  |  |
| Q08 Diff hearing even with aides | .468 |  |  |  |  |  |  |  |  |
| Q12 Slower learning/need encouragement | .414 |  |  |  |  |  |  |  |  |
| Q26 Angry/want to fight |  | .545 |  |  |  |  |  |  |  |
| Q27 Mood Swings |  | .512 |  |  |  |  |  |  |  |
| Q25 Prefers to be alone |  | .482 |  |  |  |  |  |  |  |
| Q18 Think others want to hurt him/her |  | .432 |  |  |  |  |  |  |  |
| Q24 Upset/angry with change |  | .428 |  |  |  |  |  |  |  |
| Q28 Need to move around |  | .404 |  |  |  |  |  |  |  |
| Q19 See/hear things not there |  |  |  |  |  |  |  |  |  |
| Q22 Afraid with touched or hear noise |  |  |  |  |  |  |  |  |  |
| Q31 Feel afraid for no reason |  |  |  |  |  |  |  |  |  |
| Q23 Feel better with self-injury |  |  |  |  |  |  |  |  |  |
| Q33 Fainted |  |  | .765 |  |  |  |  |  |  |
| Q34 Seizures/convulsions |  |  | .615 |  |  |  |  |  |  |
| Q35 Bitten tongue/frothed at mouth |  |  | .525 |  |  |  |  |  |  |
| Q17 Behaves in strange manner |  |  |  | .605 |  |  |  |  |  |
| Q20 Talk aloud/conversation with self |  |  |  | .431 |  |  |  |  |  |
| Q30 Unaware when spoken to |  |  |  |  |  |  |  |  |  |
| Q29 Does not feel worry/sad |  |  |  |  |  |  |  |  |  |
| Q21 Repeat same gesture continuously |  |  |  |  |  |  |  |  |  |
| Q13 Acts/behave younger than age |  |  |  |  |  |  |  |  |  |
| Q11 Later talking compared to peers |  |  |  |  | .751 |  |  |  |  |
| Q10 Later walking compared to peers |  |  |  |  | .747 |  |  |  |  |
| Q04 Diff. walking around/climbing |  |  |  |  |  | .677 |  |  |  |
| Q03 Unable to move part/entire body |  |  |  |  |  | .608 |  |  |  |
| Q06 Needs assistance with ADL’s |  |  |  |  |  |  | .580 |  |  |
| Q02 Partial or total paralysis |  |  |  |  |  |  | .472 |  |  |
| Q07 Diff. seeing even with glasses |  |  |  |  |  |  |  |  |  |
| Q16 Diff. concentrating/recall things |  |  |  |  |  |  |  |  |  |
| Q05 Bodypart looks diff. than other people |  |  |  |  |  |  |  |  | .478 |
| Q01 Lack or missing part of limb |  |  |  |  |  |  |  |  |  |
